# Molecular Subtypes and Mechanisms of Breast Cancer: Precision Medicine Approaches for Targeted Therapies

**DOI:** 10.3390/cancers17071102

**Published:** 2025-03-25

**Authors:** Eduarda Carvalho, Sule Canberk, Fernando Schmitt, Nuno Vale

**Affiliations:** 1 PerMed Research Group, RISE-Health, Faculty of Medicine, University of Porto, Alameda Professor Hernâni Monteiro, 4200-319 Porto, Portugal; ecferreira@med.up.pt (E.C.); sulecanberk@med.up.pt (S.C.); fschmitt@med.up.pt (F.S.); 2 RISE-Health, Department of Pathology, Faculty of Medicine, University of Porto, Alameda Professor Hernâni Monteiro, 4200-319 Porto, Portugal; 3 RISE-Health, Department of Community Medicine, Health Information and Decision (MEDCIDS), Faculty of Medicine, University of Porto, Rua Doutor Plácido da Costa, 4200-450 Porto, Portugal

**Keywords:** breast cancer, hormone receptor, HER2, combination therapies, precision medicine

## Abstract

Breast cancer remains one of the most prevalent diseases worldwide, primarily affecting women. Its heterogeneous nature poses a significant challenge in the development of effective and targeted treatments. Molecular characterization has enabled breast cancer to be classified into four main subtypes: luminal A, luminal B, HER2-positive, and triple-negative breast cancer, based on hormone receptor expression and HER2 status. A deeper understanding of these molecular markers and their associated signaling pathways, such as MAPK and PI3K/AKT, is essential for improving prognosis and optimizing treatment strategies. Currently, several therapeutic agents are utilized in neoadjuvant and adjuvant therapies, often in combination with surgical interventions. However, emerging evidence highlights the growing challenge of drug resistance, which significantly limits the efficacy of existing treatments. Addressing this issue may require innovative approaches, including combination therapies and precision medicine strategies, tailored to the molecular profile of each patient. Therefore, a comprehensive understanding of the pathophysiologic mechanisms driving breast cancer progression and resistance is crucial for the development of advanced targeted therapies with greater precision and efficacy. This review aims to explore recent advancements in molecular research related to breast cancer subtypes and provide a critical analysis of current therapeutic approaches within the framework of precision medicine.

## Simple Summary

This review focuses on breast cancer, a common disease that remains difficult to treat due to its heterogeneity and resistance to therapy. Here, we aim to examine the different molecular subtypes of breast cancer and how their differences impact treatment success. Moreover, we highlight that combining therapies, rather than using a single drug, can be more effective in overcoming resistance and reducing the risk of cancer returning. By analyzing the biological characteristics of tumors using advanced techniques like single-cell sequencing, this study aims to elucidate the benefits of personalized treatment strategies. The findings contribute to the medical community by promoting precision medicine, offering insight into why some treatments fail and suggesting new ways to enhance cancer therapies.

## 1. Introduction

**Figure 1:**
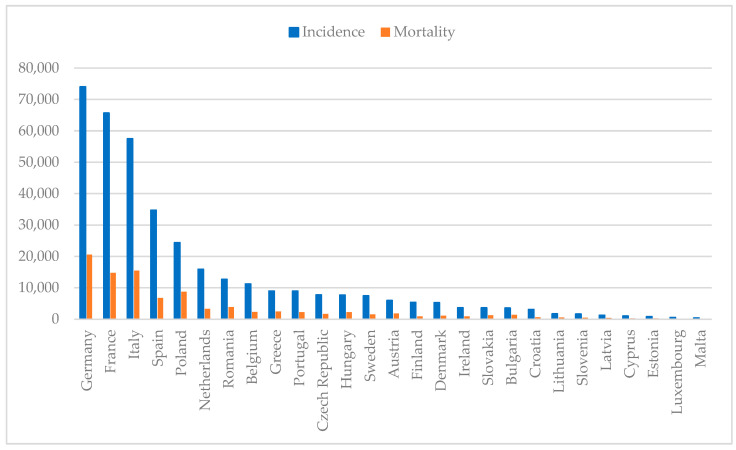
Estimated breast cancer numbers of incidence and mortality in European countries per 100,000 population in 2022 (developed from 1–3).

Breast cancer remains the most common malignancy among women worldwide, affecting 2.3 million individuals and causing 670,000 deaths in 2022 alone. With an incidence rate of 8954 per 100,000 women in Portugal, as shown in Figure 1, this disease continues to pose a major public health [1,2,3]. Despite depending on the gender, both incidence and mortality rates depend on age, ethnicity and geographics. Typically, women with African descent have lower incidence but higher mortality rate in comparison to Caucasian women, which can be attributed to differences in the access of healthcare treatments [4]. Comparing countries with high, moderate and low income, the first have a higher incidence of breast cancer than low-income countries, possibly due to better screening programs that allow cancer to be detected earlier. On the contrary, countries with low income have a higher mortality rate due to the lack of screening programs and access to healthcare [5].

The spectrum of risk factors associated with this disease is wide and diverse, going from genetic to environmental factors. Although gender and ethnicity are major risk factors for breast cancer, age is considered as one of the most important. Studies indicate a higher incidence of breast cancer in older women ranging from 55 to 64 years [4,6,7]. Respectively, new guidelines were approved to start the screening programs earlier than 50 years [8,9]. Younger women present a glandular-fibrous tissue which have a radiopaque appearance on a mammogram making more difficult to detect microcalcifications, while older women have more fatty tissue, which facilitates detection [7,10]. Studies demonstrated that as women grow older, tissue morphology changes and becomes fattier, and denser breast tissue poses a higher risk of developing breast cancer [11,12]. For these women, the guidelines are changing, and they are now eligible for magnetic resonance imaging (MRI) as strong data have emerged supporting a reduction in breast cancer mortality [13,14].

Furthermore, the personal and family history of breast cancer poses an increased predisposition for developing breast cancer. The personal history of breast cancer represents an increased risk of 2- to 6-fold of having breast cancer recurrence and the second malignancy may appear in a different breast as the first one or in the same [10,15]. Family history of breast cancer is responsible for 5% to 10% of breast cancer cases and studies have shown that women with blood relatives with breast cancer have a higher risk of developing breast cancer [16]. The same study demonstrated that women with two or more relatives diagnosed with breast cancer under the age of 45 are more susceptible to develop breast cancer [16,17]. In line with this, genetic factors play an important role in the predisposition of breast cancer. This is often related to inherited genetic variations that besides increasing the risk of cancer on their own, contribute to an increased familial risk [18]. The most common mutations studied and associated with breast carcinogenesis are BRCA1 and BRCA2 mutations, which increase the risk of cancer up to 40% [19]. These genes are known to regulate important mechanisms concerning cell growth and DNA damage repair; thus, a mutation in BRCA1 and BRCA2 is often related to genomic instability, as cells continue to proliferate despite having DNA damage, which promotes breast cancer [20]. Other mutations, such as TP53 and PTEN genes, also contribute to cell cycle irregularities and DNA repair dysregulation [21].

During a women’s reproductive years, she undergoes estrogen and progesterone fluctuations, increasing the risk of breast cancer. The more variations, the higher the risk, and this is largely explained by an early menarche and a late menopause [22]. As estrogen increases cell growth and proliferation, there is a higher probability of DNA replication errors and a reduction in DNA repair that result in cumulative alterations passed down into daughter cells that facilitate breast cancer development [23]. On the contrary, pregnancy and breastfeeding are considered protective factors of breast cancer; however, later pregnancy after the age of 35 is associated with increased risk [24,25,26]. Moreover, exogenous estrogen derived from hormonal replacement therapies or from oral contraceptives has been associated with an increased risk of breast cancer. Regarding the administration of hormonal replacement therapies, there is still some contradictory findings on whether it has a protective effect or if contributes to a higher risk of breast cancer [23,27]. A recent study demonstrated that women who were administered menopausal hormonal therapy have a higher risk of developing cancer in comparison to those that never used this therapy and revealed that the combination of estrogen with other molecules presents a higher risk than the administration of estrogen alone [28]. In addition, the usage of oral contraceptives also prolongs the exposure to estrogen, increasing the susceptibility for breast cancer development. Studies indicate that women that use oral contraceptives before the first full-term pregnancy and for more than 5 years have a significant increased risk of breast cancer [29,30].

Some environmental and lifestyle risk factors, including obesity, alcohol consumption, tobacco smoke and physical inactivity, enhance the risk of breast cancer. After menopause, the primary source of estrogen production is adipose tissue; therefore, obesity (body mass index > 30 [31]) represents a major risk factor for breast cancer [32]. The elevated levels of this hormone promote rapid cell proliferation and the overcoming of damage checkpoints, enabling mutations and DNA damage to be transmitted to daughter cells as cells have less time to correct the errors [33]. An increased alcohol consumption is associated with an increase in breast cancer risk [34]. It elevates estrogen levels in the blood, which triggers estrogen-related pathways and potentiates estrogen receptors’ production [35]. On the other hand, tobacco smoking is a major cause of several cancers [36], but there is still a controversial debate regarding its carcinogenic impact on breast cancer [37]. Recently, some studies indicated that tobacco smoking promotes breast cancer on premenopausal women, whereas it has no impact on postmenopausal women [38,39]. This may be explained by the increased estrogen levels in premenopausal women that potentiate the carcinogenic activity, while in postmenopausal women, estrogen levels decrease, thereby favoring anticarcinogenic effects [40]. Alongside these modifiable factors, physical inactivity is also associated with increased breast cancer risk [41]. A sedentary lifestyle together with a diet rich in fats contributes to obesity, which promotes breast cancer [42].

Recently, work shifts have been receiving new attention due to recent findings on increasing the risk of breast cancer development [43,44]. Working shift rotations disrupt the circadian rhythm and cause daily activities alterations [45]. The circadian rhythm works as a biological clock in the recognition of the dark–light cycle through the secretion of the melatonin hormone, helping to control the sleep cycle [46]. Findings suggest that melatonin exerts an inhibitory effect on estrogen levels; thus, an exposure to light during nighttime causes women who work night shifts to produce more estrogen than women who do not, increasing the breast cancer risk [47,48,49]. In addition, some studies point out that intensity, frequency and type of shift rotation may increase the risk [45,46,50].

Breast cancer classification is based on both morphological and molecular characteristics. Morphologically, tumors are categorized by their degree of invasiveness and site of origin within the breast tissue. Non-invasive breast cancer is confined to the epithelial structures of the mammary ducts or lobules, without invasion of the surrounding stroma [51]. This category includes ductal carcinoma in situ (DCIS) and lobular carcinoma in situ (LCIS), with DCIS representing neoplastic proliferation within the ductal system and LCIS involving lobular epithelium without stromal invasion [52,53].

In contrast, invasive breast carcinomas exhibit stromal infiltration and have the potential for regional and distant dissemination [54]. The most prevalent histotype, invasive ductal carcinoma (IDC), arises within the ducts and infiltrates adjacent breast parenchyma, often progressing to lymphovascular or systemic metastases [55]. Other subtypes include invasive lobular carcinoma (ILC), characterized by a diffuse growth pattern due to loss of E-cadherin expression [56], and inflammatory breast cancer (IBC), an aggressive variant in which dermal lymphatics are obstructed by neoplastic emboli, leading to erythema, peau d’orange and rapid progression [57]. It is noteworthy that 99% of breast carcinomas arise from ductal–lobular transition and the difference between IDC and ILC is mainly morphological and genetic. In addition, there are other types of breast cancer that are less common, such as metaplastic carcinoma, apocrine carcinoma, mucinous carcinoma, cribriform carcinoma, tubular carcinoma and neuroendocrine carcinoma [58,59].

Beyond morphological classification, molecular subtyping—based on hormone receptor status and human epidermal growth factor receptor 2 (HER2) expression—has become fundamental in guiding treatment strategies and prognostication. Advancements in molecular techniques have allowed researchers to subgroup different breast cancers, giving rise to luminal A, luminal B, HER2-positive and triple-negative breast cancer (TNBC) [60]. Luminal A refers to the presence of both hormone receptors, such as estrogen receptors (ER) and progesterone receptors (PR) in tumors; thus, this subtype is often called ER-positive. On the other hand, luminal B presents ER and may express PR, in addition to possibly expressing HER2 receptors and has a higher proliferation index than luminal A. HER2-positive breast cancer is characterized by the absence of hormone receptors and the presence of only HER2 receptors, and TNBC does not express any of these receptors, being the most aggressive subtype. This new categorization provides a new insight into the disease phenotype and prognosis, as well as guiding physicians in treatment options [59,61].

After breast cancer classification, tumor characteristics and biological behavior are assessed, helping physicians select the best treatment option among the existing ones including surgery, chemotherapy, radiotherapy, immunotherapy, hormone therapy and targeted therapies [62,63,64]. Nevertheless, the different characteristics of the tumor within the same patient (intra-heterogeneity) and between patients (inter-heterogeneity) along time and space (temporal and spatial heterogeneity) represent one of the biggest pitfalls in breast cancer treatment [65]. Intra-tumor heterogeneity refers to the presence of diverse cancer cell populations within the same tumor with distinct genetic, molecular and phenotypic aspects and it is often seen between the primary tumor and the metastatic lesion [65,66], while inter-tumor heterogeneity pertains to the difference between tumors in different patients driven by genetic mutations, molecular subtypes and hormone status [65]. In addition, genetic and molecular variations within the primary tumor or between the primary tumor and metastatic lesions across time and depending on the presence of therapy contribute to spatial and temporal heterogeneity to the tumor [67]. The manipulation of tumor genetics and epigenetics contributes to different heterogeneities, causing phenotypic alterations that may lead to therapy resistance [68,69].

Indeed, drug resistance poses a major challenge for breast cancer therapy, and it may lead to patient relapses. It can be acquired, and it is only detected after several failed treatment administrations, or it can be intrinsic and the therapy does not improve the patient’s survival [70]. Even though therapy resistance has been a problem in traditional therapies, it has been an emerging challenge in targeted therapies that target specific molecules and key signaling pathways due to epigenetic alterations, tumor heterogeneity and microenvironment, enhanced DNA repair and drug efflux [71,72]. Sometimes, this leads to treatment persistence or overtreatment resulting in patient toxicity and multiple side effects. Chemotherapy and radiotherapy usually lead to neurotoxicity, cardiotoxicity and nephrotoxicity besides encompassing side effects on the gastrointestinal and genitourinary tracts, lungs and skin, while targeted therapies possess a selective toxic profile [73,74,75].

To address these challenges, recent findings demonstrate that personalized and combinatorial approaches are the best methods to overcome it [76,77,78]. Therefore, the aim of this work is to provide an insight into the molecular mechanisms of breast cancer molecular subtypes and to enlighten the efficacy of novel personalized treatments of this disease over traditional therapy.

## 2. Types of Cancer Based on Molecular Classification

Recent advances in molecular technologies have facilitated breast cancer research, allowing researchers and physicians to use biomarkers expressed by cancerous cells to categorize this pathology into four distinct groups—luminal A, luminal B, HER2-positive and TNBC. Thereby, the main molecular markers used are ER, PR and HER2, and they are detected using immunohistochemical techniques. In addition to these markers, Ki-67 is a widely used biomarker of tumor proliferation that helps complement and understand the pathology as demonstrated in Table 1. Together, they provide insights on diagnosis, prognosis and treatment [79].

The hormone receptors ER and PR are associated with the female reproductive system. Tumor cells that are ER-positive (≥1%) are defined as luminal A breast cancer. In addition, this type of cancer expresses high levels of PR (≥20%) and presents a low expression of Ki-67 (<20%), indicative of low tumor proliferation. Consequently, among the several types of cancer, this is the least harmful and the most common, being responsible for 50–60% of cases [59,80,81]. Furthermore, breast cancers categorized as IDC and ILC, mucinous, cribriform and tubular carcinomas fall under the subtype of luminal A breast cancer [59]. Luminal B breast cancer expresses the hormone receptor ER (≥1%) and it can be HER2-negative (≤10%) or HER2-positive (>10%). Usually, if luminal B is HER2-negative, the expression of PR is low to none (<20%) and the biomarker Ki-67 is highly expressed (≥20%), but if the luminal B cancer is HER2-positive, the levels of PR and Ki-67 varies [59,81]. However, it often presents higher levels of Ki-67 than luminal A, accelerating tumor growth and being associated with worse prognosis [80,81].

HER2 is an onco-protein that upon a strong stimulus starts to divide uncontrollably and, as a response, the mammary cells produce an excessive amount of HER2 receptors. This overexpression of HER2 gives rise to HER2-positive breast cancer, which is characterized by the absence of hormone receptors (ER < 1% and PR < 20%) and the presence of high levels of HER2 (>10%) [59]. This subtype of cancer is further subdivided into three subgroups, namely, HER2-positive if the score of immunohistochemistry is 3+ or if the score is 2+ and in situ hybridization is positive, HER2-low if the score of immunohistochemistry is 2+ and the in situ hybridization assay is negative or if the score of immunohistochemistry is 1+, and HER2-negative if the score of immunohistochemistry is 0 [82]. The HER2-enriched breast cancer is typically associated with ILC, and it is responsible for 10–15% of all breast cancers, presenting a high expression of Ki-67 (>20%), which makes this cancer the one with worse prognosis and with the fastest pace of growth in comparison to luminal breast cancer [79,80,81].

TNBC is defined by the absence of hormone receptors (ER < 1% and PR < 20%) and HER2 (≤10%) and represents 10% of all breast cancer cases. It is characterized by DNA alterations and genetic mutations, being mostly associated with mutations in the BRCA1 gene [59]. It is the most aggressive among the four subtypes of breast cancer with the highest proliferation index of Ki-67 (>30%) and poorer prognosis, and it is usually related to IDC and variants of apocrine and metaplastic carcinomas [59,80,81]. Furthermore, several studies of gene expression revealed that TNBC can be divided into seven different subtypes, including basal-like 1 (BL1), basal-like 2 (BL2), mesenchymal-like (M), mesenchymal stem-like (MSL), immunomodulatory (IM), luminal androgen receptor (LAR) and claudin-low type (CL) [59,83].

**Table 1:** Molecular characteristics and diagnostic guidelines of molecular subgroups of breast cancer.

Types of Cancer	Subgroups	Biomarkers	Other Markers	Diagnostic Guidelines	Reference
Luminal	Luminal A	ER (≥1%), PR (≥20%), Ki-67 (<20%)	BCL2, CK8/18, ESR1, GATA3, TRPS1, GPR160, TMEM45B, BAG1	[84]	[85,86,87]
	Luminal B (HER2-negative)	ER (≥1%), PR (<20%), HER2 (≤10%) Ki-67 (≥20%)	CK8/18, GATA3, TRPS1, ESR1, FOXA1, CXXC5, BIRC5, BLVRA, UBE2T		[86,87]
	Luminal B (HER2-positive)	ER (≥1%), PR (<20%), HER2 (>10%), Ki-67 (15–30%)	CK8/18, GATA3, TRPS1, MKI67, cyclin B1		[85,86]
HER2-enriched	HER2-positive	ER (<1%), PR (<20%), HER2 (>10%), Ki-67 (>30%)	ERBB2, MYC, MAPK, TP53, VHL, ATM	[88]	[89,90]
	HER2-low	ER (≥1%), HER2 (>10%), Ki-67 (≤30%)	BCL2, BAG1, FOXA1, ESR1, CCNE1, CCNB1, MYBL2, MKI67, MELK		[91,92]
TNBC	BL1	ER (<1%), PR (<20%), HER2 (≤10%), Ki-67 (>30%)	EGFR, CK5/6, CK14, CK17, MYC, PIK3CA, CDK6, AKT2, KRAS, FGFR1, IGF1R, CCNE1, CDKN2A/B, BRCA, PTEN, MDM2, RB1, TP53	[84,88,93]	[83,94]
	BL2		EGFR, MET, NGF, IGF-1R, Wnt-β-catenin		[83]
	M		Wnt, TGF-β, DNMT3A, TP53, PDGFRA, MAP3K1		[83,94]
	MSL		ABCA8, PROCR, ENG, ALDHA1, PER1, ABCB1, TERT2IP, BCL2, BMP2, THY, HOXA5, HOXA10, MEIS1, MEIS2, MEOX1, MEOX2, MSX1, ITGAV, KDR, NGFR, NT5E, PDGFR, THY1, VCAM1		[83]
	IM		Th1/Th2, IL-12, IL-7, TP53		[83,95]
	CL		ALDH1A1, PROCR, ZEB1, VIM, CDH2, EPCAM, CDH1, IL-13, ALDH1A1, PROCR, ZEB1, VIM, CDH2, EPCAM, CDH1, IL-13, IL6, CXCL8, VEGF-C, NRF1, EREG		[96,97]
	LAR	AR (≥10%), ER (<1%), PR (<20%), HER2 (≤10%), Ki-67 (>30%)	DHCR24, ALCAM, FASN, FKBP5, APOD, PIP, SPDEF, CLDN8, PIK3CA, AKT1, NF1, GATA3, CDH1, KMT2C		[83,94,95]

## 3. Molecular Mechanisms of Breast Cancer

Genetic mutations and alterations in signaling pathways play pivotal roles in the onset and progression of breast cancer. These mutations can disrupt key regulatory processes, including cell growth, survival, proliferation, apoptosis and DNA repair, leading to tumorigenesis. In particular, the crosstalk between genetic alterations and dysregulated signaling pathways contributes to the heterogeneity observed across the different breast cancer subtypes [98]. Understanding the molecular mechanisms underlying these mutations is critical for identifying therapeutic targets and improving patient outcomes.

### 3.1. Luminal Breast Cancer

Estrogen belongs to a group of steroid hormones, and it is associated with the female reproductive system. There are four distinct forms of estrogen including estrone (E1), estradiol (E2), estriol (E3) and estetrol (E4) [99]. E1 levels increase after menopause and have a pro-inflammatory effect in breast and adipose tissue, suggesting it is responsible for poor outcomes of ER-positive breast cancer in obesity [100]. The E2 form of estrogen is the most common in human blood circulation and it is produced by granulosa cells present in the ovaries [99,101]. E3 and E4 are both produced during pregnancy. Whereas E3 is synthesized by the placenta, E4 is a byproduct secreted by the fetus’ liver and they are both transported into the mother’s circulation through the placenta [101,102].

Estrogen, primarily E2, exerts its function by binding to two different ER, namely, ERα and ERβ, which are nuclear transcription factors involved in the regulation of many biological processes [101]. Mutations in these receptors have a great impact in cancer cells proliferation [103].

The receptor ERα plays a crucial role in cancer development through the regulation of target genes related to cell proliferation, apoptosis and cell cycle [80]. This receptor is activated upon E2 binding, causing a change in its configuration into an active form, dimerization and subsequent translocation to the nucleus. Through this mechanism, estrogen can regulate the expression of many estrogen-responsive elements (ERE) by binding to their gene promotor region instead of binding directly to DNA [80,104]. Once the estrogen is bound to EREs, ER recruits co-activators to assist the transcription of target genes. However, a dysregulation in ERα expression causes an unbalance between Ap1 and SP1 transcription factors and coregulators of target genes, as SRC1, AIB1, NCOR and MTA1, playing a major role in the development of luminal breast cancers [80,105,106].

Additionally, E2 can also bind to ERs present in the plasma membrane, activating the plasmatic ERα which attaches to caveolin-1, thus inducing downstream signaling pathways including PI3K/AKT/mTOR, a major pathway typically involved in cell growth, metabolism, proliferation, apoptosis and angiogenesis [80,107].

Under normal conditions, after the activation of ERα, E2 directly interacts with the p85 subunit of PI3K, activating the p110 catalytic subunit that promotes the phosphorylation of PIP2 into PIP3 which in turn recruits AKT. Following this, the AKT is phosphorylated by mTOR complex 2, changing its conformation into an active form that promotes the phosphorylation of target proteins present in the cell membrane, cytosol and in the nucleus. However, in breast cancer, PI3K suffers many changes due to genetic alterations and amplifications that are responsible for key products that ensure the normal signaling pathway. The catalytic subunit p110α regulated by the PIK3CA gene of PI3K is mutated, causing an hyperactivation of this gene, while there is a loss of PTEN, resulting in tumor growth. This overactivation of the signaling pathway PI3K/AKT/mTOR increases cell proliferation and inhibits apoptosis, enhancing breast cancer development [103,107].

On the other hand, ERβ is often co-expressed with ERα, expressing similar transcription factors and co-activators, but it plays an antagonistic role in comparison to ERα [80]. Studies find that ERβ acts as a tumor suppressor by inhibiting its proliferation, reduces inflammation and induces apoptosis of breast cancer cells, aiding in breast cancer therapy and being associated with better prognosis [108,109]. Recent publications support this mechanism, stating that ERβ has an anti-proliferative effect by downregulating the expression of ERα, diminishing epithelial–mesenchymal transition, which reduces cell invasion, and induces the entering in G2 phase arrest that promotes the inhibition of tumor growth [110,111].

Progesterone belongs to the same group of steroid hormones of estrogen and is responsible for the regulation of female pregnancy, menstrual cycle and embryogenesis. In addition, it promotes mammary epithelial cells proliferation and mammary gland expansion [112].

In breast cancer, progesterone signaling provokes a switch from paracrine regulation to autocrine regulation, being a hallmark of tumor progression. A progesterone-derived hormone named hormone medroxyprogesterone acetate turns epithelial cells more susceptible to breast tumors by triggering the nuclear factor-κB (NF-κB). Therefore, the progesterone binds to PR, activating this receptor, which results in an upregulation of RANKL, thereby increasing the expression of RANK receptor [112,113]. Other studies support this finding, as an increase of RANKL correlates with breast cancer development, which is also associated with poor prognosis in patients with luminal B breast cancer [114]. Hence, this pathway plays a major role in mammary tumorigenesis in response to progesterone. Nevertheless, PR has two isoforms, PRA and PRB, and the ratio between these two significantly influences breast cancer progression. It has been demonstrated that an increase in PRA is related to breast cancer development and metastasis, while an increase in PRB correlates with tumor proliferation [114]. The role of progesterone in breast carcinogenesis has not yet been clarified; therefore, further research needs to be conducted to enlighten the mechanism of PR in breast cancer.

### 3.2. HER2-Positive Breast Cancer

HER2 is a tyrosine kinase receptor and is a part of the epidermal growth factor receptor (EGFR) family. Commonly, its structure presents an intracellular tyrosine kinase that regulates genes involved in several cellular functions, a membrane domain that transmits the signal to the nucleus and an extracellular component where ligands, namely, HER1, HER3 and HER4, bind to their respective receptors and trigger a specific signal [115]. However, unlike the hormone receptors ER and PR, HER2 does not have ligand-dependent activity and thus depend on other family members to heterodimerize or homodimerize with itself to be activated. This phosphorylation of tyrosine kinases induces a conformational change on the intracellular component that promotes the activation of secondary signaling pathways, such as PI3K/AKT and MAPK [115,116].

Typically, phosphorylated HER2 and dephosphorylated HER2 are at an equilibrium, which is responsible for normal breast development. Upon a strong oncogenic stimulus, phosphorylated HER2 becomes upregulated, disrupting the balance that causes cell invasion, differentiation and migration, which are common features of tumor development and progression [117]. Thus, the amplification of HER2 is a hallmark of HER2-positive breast cancer that provokes hyperactivation of the signaling pathways, contributing to a more aggressive biological behavior and worse clinical outcomes [115,116]. However, recent findings have suggested a new subtype of HER2-positive breast cancer called HER2-low breast cancer [82]. Several studies were performed to understand the molecular biology of HER2-low subgroup of breast cancer, and it was predominantly associated with luminal A and luminal B cancer [118]. Nevertheless, a clear definition of this condition has not yet been formulated; thus, further investigations are needed to better understand it and optimize treatments for eligible patients [119].

When HER2 heterodimers with HER3, it interacts with the p85 subunit of PI3K activating the catalytic subunit p110. In HER2-positive breast cancer, the HER2 overexpression causes cellular signaling stress and increases DNA replication errors, resulting in mutations in the PIK3CA and AKT genes of the PI3K/AKT signaling pathway. Moreover, PTEN acts as a negative regulator of PI3K/AKT, maintaining it inactive and keeping the balance between cell survival and apoptosis. However, in cancerous conditions, this regulator is dysfunctional and thus promotes the activation of this pathway. Even though PTEN deletion is more correlated to poor outcomes in ER-positive breast cancer, its conjugation with PIK3CA and AKT gene mutations is associated with mammary tumor initiation and proliferation [115,117]. In addition, findings assessing the molecular landscape of HER2-positive breast cancer patients reported an elevated frequency of PIK3CA mutations (61.5%), but the difference to PIK3CA wild-type was not significant [120]. This indicates that this pathway may not be the main player in the development of this breast cancer subtype.

Moreover, the MAPK signaling cascade is also a major player in breast cancer. The aberrant overexpression of HER2 recruits more growth factors receptor-bound proteins 2 (GRB2) to be activated, which, together with the SOS, facilitates the exchange of guanosine diphosphate (GDP) into guanosine triphosphate (GTP) that activates RAS [121]. This hyperactivation of RAS causes a dysregulation in the activation of downstream protein kinases by promoting the phosphorylation and dimerization of RAF, which is strongly associated with carcinogenic conditions. Furthermore, the kinases RAF and RAS form a heterodimer that activates RAF, which phosphorylates MEK1 and MEK2 that, in turn, triggers the activation of ERK1 and ERK2. These activated ERK translocate to the nucleus, where they regulate several transcription factors including ETS1/2, ELK-1 and JUN involved in tumor proliferation, resistance to apoptosis and enhanced metastasis that contribute to cancer progression [121,122,123].

### 3.3. Triple-Negative Breast Cancer

TNBC does not express any hormone receptor or HER2 receptors and often arises from genetic alterations and mutations, causing an extended genomic heterogeneity. The BRCA1 and BRCA2 genes play a critical role in DNA repair by homologous recombination pathways or by influencing the cell to enter in non-homologous end joining pathways; they activate important cell cycle checkpoints to assess DNA damage and ensure that it does not present any abnormality; and they participate in cytoplasmic division and induce cell apoptosis and ubiquitination when the damage on the DNA cannot be repaired by inhibiting oncogenes amplification [124,125]. Typically, mutations in these genes are inherited from a relative that has this genetic mutation in their germinative cells and are related to the appearance of cancerous indicators at a young age, but mutations can also be acquired from several environmental factors that cause DNA damage. Mutations in somatic cells are not passed on to the offspring [124,126,127].

BRCA1 mutation inhibits the homologous recombination pathway, leading to the activation of alternative DNA damage repair pathways, while BRCA2 mutations compromise cytoplasmic division, increasing the number of binucleated cells and provoking chromosomic alterations which leads to aneuploid cells [127,128]. Taken together, they contribute to genomic instability and subsequent tumor initiation and progression. It has been stated that patients with BRCA1 gene mutations also present TP53 mutations [129] and several cohorts that studied the genetic profile of patients with TNBC proved this to be true. A study conducted with 95 female patients that have been submitted to primary surgery or neoadjuvant chemotherapy demonstrated that among the patients that suffered from TNBC, TP53 was the most frequent mutation (30%), followed by BRCA1 gene mutation (13%) and BRCA2 mutation (7%). Beyond this, they verified that BRCA2 mutations (16%) were more prevalent in luminal breast cancers in comparison to the other two genes and that TP53 mutations (18%) were also more common in HER2-positive breast cancer [130]. After assessing the prognostic value of each gene mutation, they concluded that TP53 and BRCA1 are the best prognostic biomarkers in TNBC being associated with worse outcomes. Another cohort with 52 patients with TNBC studied the mutation of genes related to DNA damage repair pathways, such as BRCA1, BRCA2 and TP53, among others. It was found that 75% of the patients presented BRCA1 mutations against 25% that presented BRCA2 mutations. When assessing the non-BRCA mutations landscape, they reported TP53 mutations in almost all the patients, and although BRCA1 was more prevalent than BRCA2 mutations, it was noteworthy that PIK3CA was more commonly co-mutated with the latter [131]. This last finding suggests that in TNBC, the PI3K/AKT/mTOR signaling pathway also suffers alterations affecting key cellular regulatory processes. An older study from 2022 with a larger sample composed of 42,680 female patients with diagnosed breast cancer from several countries associated TP53 mutations with HER2-positive breast cancer, but not with TNBC, while BRCA1 and BRCA2 variants were strongly present in this latter condition [132]. This is in line with what was recently found when comparing genetic mutations between the different subtypes of breast cancer.

Alterations in the PI3K/AKT/mTOR signaling pathway result in a disruption of regulatory cellular processes; therefore, PIK3CA mutations increase tumor cells proliferation, migration and invasion, as well as resistance to apoptosis [133]. Numerous studies comparing PIK3CA mutations in hormone receptor-positive breast cancer against TNBC have demonstrated that this mutation is less prevalent in the most aggressive subtype [133]. Mosele F. et al. found that 10% of TNBC cases reported PIK3CA mutations against 28% in hormone receptor-positive breast cancer [134]. Consistent with this finding, a study with 1270 female patients with breast cancer showed that PIK3CA mutations are more prevalent in hormone receptor-positive breast cancer, especially with HER2-negative status (31.4%) in comparison to TNBC (11.7%) [135]. Therefore, TNBC is more affected by mutations in genes involved in DNA repair than by PIK3CA mutations. Impairment of the machinery involved in DNA reparation promotes the propagation of mutations throughout the cell division, which contributes to uncontrollable tumor cell growth and genetic instability conferring the aggressive nature of this cancer subtype.

## 4. Therapeutic Approaches

Breast cancer treatment has evolved over the past century, transitioning from surgeries to personalized medicine. Technological advances have allowed for a better understanding of medical science and a deeper knowledge of cancer biology, which paved the way for a multidisciplinary approach that integrates surgery, chemotherapy and targeted therapies. Today, molecular profiling and disease subtyping guide treatment strategies, ensuring that therapies are tailored to each patient to improve survival rates. Table 2 summarizes the main findings and conclusions of the studies mentioned in each therapy.

### 4.1. Endocrine Therapy

**Figure 2:**
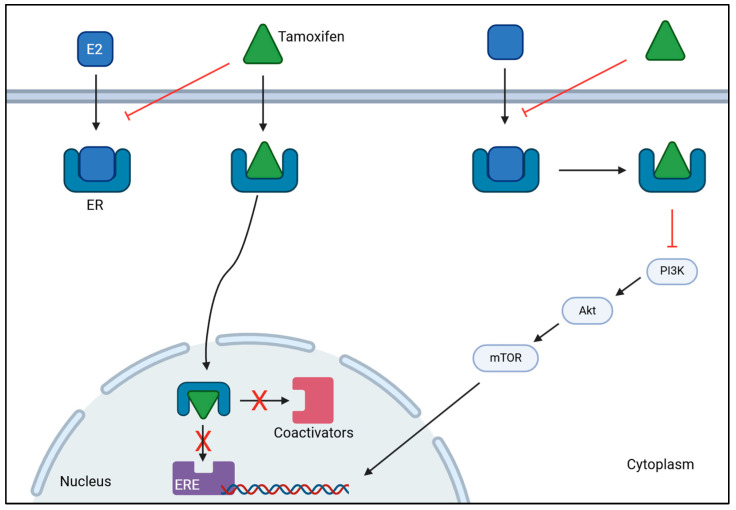
Mechanism of action of tamoxifen on ER-positive breast cancer. Tamoxifen binds to estrogen receptors (ER), preventing estrogen (E2) from binding. This inhibits the activation of estrogen-responsive elements (ERE) and the subsequent recruitment of coactivators, stopping gene transcription. Tamoxifen can also bind to estrogen receptors to inhibit the activation of PI3K/AKT/mTOR signaling cascade to decrease the transcription of genes involved in tumor progression. Together, they hamper cancer proliferation and progression. [BioRender, 2025].

Endocrine therapy is the treatment of choice to manage luminal breast cancer, specifically ER-positive breast cancer. This strategy directly targets ERs to prevent ligands from binding to it, and inhibits ovarian estrogen production, which promotes a reduction in estrogen levels [136,137]. To this end, various drugs are used including tamoxifen (TAM) and aromatase inhibitors (AI), such as anastrozole, letrozole and exemestane [138,139]. TAM is a selective estrogen response modulator (SERM) and is mostly used in patients with breast cancer who cannot endure AI [140]. This drug acts as an ER blocker by binding to ERα of breast cancer cells, which prevents estrogen from binding to the receptor. This mechanism of action provokes estrogen inactivity by suppressing transcriptional action or by reducing its effect, thereby stopping the onset and progression of breast cancer as illustrated in Figure 2 [140,141]. AI works by blocking the aromatase enzyme, preventing the production of E2 from androgen’s conversion or the synthesis of E1 from testosterone’s conversion, which inhibits its action on ER, thus decreasing estrogen levels [142,143].

Since ER-positive breast cancer development depends on estrogen, these inhibitors are particularly effective in treating breast cancer in postmenopausal women [144]. A study with 31,920 women with ER-positive breast cancer was conducted to determine the effect of TAM treatment versus AI treatment on recurrence risk and mortality rate in a 5-year period. Although no significant differences were found between the group under TAM plus AI therapy and the group under AI monotherapy, women treated with AI had a 30% recurrence risk reduction and a 15% mortality rate reduction when treatment differed [145]. Consistent with this finding, Rosie B. et al. performed a meta-analysis in female premenopausal patients with ER-positive breast cancer receiving ovarian suppression to assess if they would benefit from AI treatment in a 5-year period. Results showed that patients treated with AI had lower recurrence risk (3.2%) of breast cancer than women who underwent TAM treatment (6.9%). Regarding mortality, they observed that patients who received AI treatment had higher mortality rates in the 0–4 period of follow-up, but the same patients had a mortality rate reduction in the following years (5–9 years) [146]. This suggests that AI is more effective in reducing breast cancer, serving as a more effective treatment than TAM by having a long-term effect on ER-positive breast cancer.

### 4.2. Anti-HER2 Therapy

**Figure 3:**
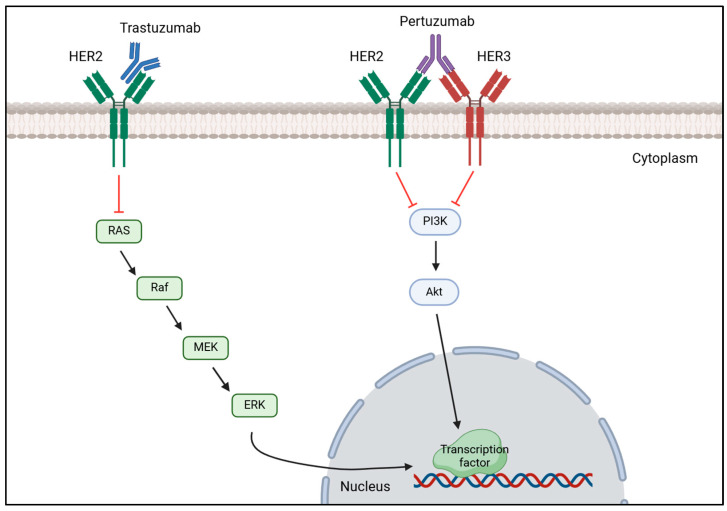
Mechanism of action of trastuzumab and pertuzumab on HER2-positive breast cancer. Trastuzumab binds to the extracellular domain of HER2 receptor to prevent it from being activated, decreasing the expression of the MAPK signaling cascade that results in a reduction of transcription factors’ transcription important for tumor proliferation. Pertuzumab binds to HER3, preventing HER2-HER3 heterodimerization, which inhibits the phosphorylation of PI3K subunits, decreasing the activation of the PI3K/AKT signaling pathway, which in turn does not activate the transcription of transcription factors important for cell proliferation. [Biorender, 2025].

Anti-HER2 therapy is the recommended treatment for patients with HER2-positive breast cancer and typically relies on pertuzumab and trastuzumab as primary treatment [138,147]. This strategy works by blocking the HER2 signaling pathway by monoclonal antibodies, such as trastuzumab, binding to HER2 receptor which prevents HER2 activation and dimerization, thereby inhibiting HER2 overexpression [148]. Likewise, pertuzumab blocks HER2 overexpression by preventing HER2-HER3 dimerization resulting in HER2 signaling pathway inhibition as it is demonstrated in Figure 3 [149]. A study conducted by Hamid M. and Zhixiang W. on the effect of trastuzumab on the development of HER2-positive breast cancer cells revealed that this drug inhibits cancer cells proliferation by promoting the entering in G1 phase arrest, but did not have any effect on HER2 dimerization [150]. Despite having less HER2, the remaining HER2 molecules continue to heterodimerize with HER3 or homodimerize with itself; thus, research has arisen combining trastuzumab and pertuzumab to completely block the proliferation of tumorous cells and improve the outcome of patients with this disease [151]. Li-Chung T. et al. tested the combination of trastuzumab and pertuzumab both in vitro and in vivo and highlighted that dual therapy maximized the therapeutic effect in comparison to monotherapy of either drug promoting tumor recognition by the immune system to destroy it, facilitating cancerous cell lysis and subsequent phagocytosis [152]. Multiple research reenforced that the usage of these two drugs together with chemotherapy would significantly improve patient outcomes and overall survival (OS) rates [153,154]. A domestic study performed in China evaluated the efficacy of dual anti-HER2 therapy using trastuzumab with pertuzumab in combination with different types of chemotherapy. After treatment administration, the overall pathological complete response (pCR) rate was 60.9% and the expression of HER2 was reduced in four patients, becoming HER2-negative and confirming the efficacy of this treatment [155]. Consistently, a muti-center study confirmed the efficacy of trastuzumab and pertuzumab with chemotherapy in reducing the recurrence risk (7.4 years versus 6.7 years) and mortality rate (7.6 years versus 7.2 years) in patients who achieved pCR [156]. Additionally, these two studies also demonstrated the effect of combinational therapy, proving that the adverse effects are tolerable and acceptable.

Moreover, HER2-positive cancer is also treated with antibody-drug conjugates (ADC) that are targeted therapies that combine cytotoxic agents with monoclonal antibodies with the purpose of minimizing the toxic side effects typically associated with chemotherapy while maximizing the efficacy of the therapy [76]. Trastuzumab emtansine (T-DM1) and trastuzumab deruxtecan (T-DXd) are the most used ADC to treat this condition. When trastuzumab binds to HER2 in the cell surface, it is internalized by endocytosis, and when emtansine is released inside the cell, it inhibits microtubule assembly and causes cell death, while deruxtecan inhibits DNA replication and repair, induces cell cycle arrest and promotes apoptosis [157,158]. Phase 3 of the DESTINY-Breast03 randomized trial compared the efficacy of T-DM1 to T-DXd in HER2-postive breast cancer patients, and individuals treated with T-DXd had higher progression-free survival (PFS) and OS rate [159]. A subgroup analysis of the patients enrolled in this study confirmed these results that patients treated with T-DXd had longer PFS and higher OS rate in comparison to individuals treated with T-DM1 [160]. Together, they highlight the benefit of using T-DXd as a secondary treatment to treat HER2-positive breast cancer.

### 4.3. TNBC Therapy

**Figure 4:**
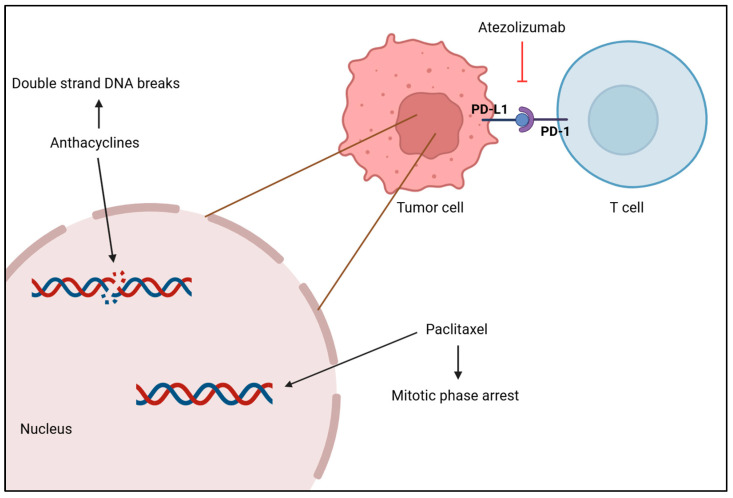
Anthracycline intercalates with DNA base pairs, causing the strand to break and leading to cell apoptosis. Paclitaxel-based chemotherapy increases the microtubule stabilization, causing the loss of their dynamic instability, important for chromosome alignment and segregation, which leads to M phase arrest and cell death. Atezolizumab inhibits PD-L1 from binding to PD-1 by binding itself to PD-L1, which promotes the T cell to recognize tumor cells again and leads to tumor cell death. [Biorender, 2025].

The standard treatment of care for TNBC is chemotherapy, but immunotherapy and radiotherapy have been attracting a lot of attention as different ways to treat this condition [161]. Systemic chemotherapy is the most common and effective treatment for TNBC, and it can be used as a neoadjuvant or adjuvant therapy. Drugs such as anthracyclines, platinum, taxanes and capecitabine are used to target and kill cancerous cells throughout the body, as seen in Figure 4, but treatment dosage should take tumor size and aggressive morphological features into consideration, as well as age and cancer stage [138,161,162]. A meta-analysis of 100,000 women from 86 randomized trials showed that adding anthracycline to a taxane-based chemotherapy regimen reduces the risk of recurrence by 14% and the annual mortality rate by about 12% in comparison to treatment with taxane monotherapy [163]. Another study assessed the effectiveness of platinum-based chemotherapy on patient’s survivability. Results showed that this treatment, both in the neoadjuvant and adjuvant setting, improved disease-free survival (DFS) and OS rates obtaining hazard ratios of 0.63 and 0.69, respectively, as a neoadjuvant therapy, and hazard ratios of 0.69 and 0.70, respectively, as an adjuvant modality. Moreover, it was reported that patients under platinum-based neoadjuvant chemotherapy had a 44% increase in achieving pCR, which is suggestive of better long-term outcomes [164]. Xi W. et al. compared the efficacy of adding capecitabine after standard adjuvant chemotherapy in early-stage TNBC between a placebo group and a capecitabine group. Research showed that maintaining capecitabine after chemotherapy improved DFS and OS, as well as locoregional recurrence-free survival by 10%, 4% and 4%, respectively. This proves that combining capecitabine with chemotherapy provides a survival benefit with tolerable adverse effects, but after a year of treatment, side effects worsen and may promote hand–foot syndrome [165].

Radiotherapy is typically used after a mastectomy to remove possible remaining tumor cells in the breast and lymph nodes [161]. A study with the aim of clarifying the role of radiotherapy in TNBC’s survival after surgery demonstrated that patients who received radiation had higher OS and better breast cancer-specific survival than patients who did not receive it, plus it reports that although it was efficient in several breast cancer stages, it was more efficient in later stages [166]. Moreover, immunotherapy has been emerging as a promising therapy for TNBC in which tumor cells are killed by the immune system, namely, by immune checkpoint inhibitors (ICI), which include cytotoxic T-lymphocyte-associated antigen 4 (CTLA-4), programmed death receptor-ligand 1 (PD-L1) and programmed death receptor-1 (PD-1) as shown in Figure 4 [167]. Atezolizumab is a monoclonal antibody that targets PD-L1 and inhibits its activity, enhancing the ability of T cells to recognize and destroy tumor cells. Research has shown that patients with PD-L1-positive tumors presented an OS higher when treated with atezolizumab plus nab-paclitaxel than patients treated with placebo plus nab-paclitaxel (25 months versus 18 months) and a longer PFS (7.5 months versus 5.3 months) [168], which highlights the clinical relevance of atezolizumab in treating patients with PD-L1-positive tumors with TNBC. Phase II of the KEYNOTE-086 study evaluated the efficacy of pembrolizumab monotherapy in patients with PD-L1-positive TNBC and observed a higher PFS within a disease-free interval higher than 12 months (2.2 months versus 2 months). Furthermore, results showed that this monotherapy improved the OS, especially when the disease-free interval was higher than 12 months (23 months versus 9.4 months), supporting the effectiveness of pembrolizumab treatment in these patients [169].

The heterogeneous nature of this disease and the lack of biomarkers poses an obstacle for the development of targeted therapies that could be tailored to each patient and would be beneficial to improve clinical outcomes. Consequently, clinicians treat every case of TNBC similarly with neoadjuvant therapy [170]. However, advances in the field show immunotherapy as a promising approach by leveraging the immune system to target tumor cells. Further research shows the beginning of the development of targeted therapies to treat TNBC and offers new therapeutic strategies for the creation of patient-tailored treatments [171,172].

**Table 2:** Main findings and conclusion of the studies involving different therapies to treat breast cancer.

Study Objective	Methodology	Main Findings	Conclusions	Reference
**Endocrine Therapy**				
Understand the benefits of using AI and TAM and the effectiveness and impact of different treatment schedules over a 5-year period	Meta-analysis of individual patient data	✓ AI is more effective over a 5-year period ✓ Undergoing a 5-year treatment of AI is slightly better than switching from TAM to AI ✓ Switching from TAM to AI is better than undergoing a 5-year treatment of TAM	Benefits were clearer when the treatment was different	[145]
Investigate if premenopausal women receiving ovarian suppression therapy benefit from AI in comparison to TAM to reduce breast cancer recurrence and improve patient survival over a 5-year period	Meta-analysis of individual patient data	✓ Reduction in recurrence in women treated with AI ✓ Risk ratio of mortality was 1.25 in years 0–4 and 0.80 in years 5–9 in AI vs. TAM	Main benefit from taking AI was best seen when patients were treated differently	[146]
**Anti-HER2 Therapies**				
Investigate the mechanisms by which the combination of trastuzumab and pertuzumab enhance antitumor activity in HER2-positive breast cancer	In vitro (BT-474, KPL-4, SKBR3, Au565, SKOV3 and NIH/3T3 cells) In vivo (SCID-beige mice and C1qa-KO mice)	✓ Monotherapy can inhibit tumor growth, but dual therapy enhances the therapeutic effect ✓ Combination of trastuzumab plus pertuzumab enhances tumor visibility to the immune system	Dual therapy of pertuzumab plus trastuzumab promotes tumor cell lysis and tumor phagocytosis by macrophages	[152]
		✓ Dual therapy promotes tumor cell phagocytosis		
Investigate if adding pertuzumab to trastuzumab and chemotherapy improves disease recurrence and reduces mortality in HER2-positive early breast cancer patients	Randomized, multicenter, multinational clinical trial	✓ Pertuzumab increased DFS ✓ Pertuzumab decreased invasive-disease events and increased invasive-DFS in hormone receptor-positive patients	The addition of pertuzumab to trastuzumab and chemotherapy increases the treatment efficacy by reducing patient relapses and mortality risk	[153]
Evaluate OS and PFS in HER2-positive metastatic breast cancer patients receiving trastuzumab plus pertuzumab or trastuzumab plus pertuzumab with chemotherapy	Multicenter randomized clinical trial	✓ Trastuzumab plus pertuzumab with chemotherapy decreased mortality and increased OS and PFS	Dual anti-HER2 therapy with chemotherapy increases OS and significantly enhances PFS	[154]
Assess the efficacy and safety of dual anti-HER2 therapy in combination with chemotherapy in HER2-positive breast cancer patients in China	Retrospective study in China	✓ Dual anti-HER2 therapy combined with chemotherapy increased pCR ✓ It promoted cell proliferation reduction ✓ Anemia was the most common side effect	Elevated pCR demonstrates the efficacy of trastuzumab plus pertuzumab with chemotherapy and the side effects are tolerable	[155]
Assess the combinatory treatment of pertuzumab plus trastuzumab and chemotherapy in HER2-positive breast cancer patients	Retrospective, multicenter study	✓ Patients who achieved pCR had higher OS and longer distant DFS	This combinatory therapy promotes higher pCR, which is linked to decreases disease recurrence and high survivability	[156]
Compare the efficacy and safety between T-DXd and T-DM1 in patients with metastatic HER2-positive breast cancer	Randomized, multicenter clinical trial	✓ PFS was higher for patients treated with T-DXd ✓ OS was higher for patients treated with T-DXd ✓ More patients treated with T-DXd achieved complete response ✓ Worse side effects of T-DXd were mostly from gastrointestinal and hematological nature	T-DXd significantly improved the OS and PFS and reduced the risk of mortality	[159]
Evaluate the efficacy and safety of trastuzumab deruxtecan compared to trastuzumab emtansine in Asian patients with HER2-positive metastatic breast cancer who were previously treated with trastuzumab and taxane	Subgroup analysis of a group of patients enrolled in the Breast03 study	✓ T-DXd prolonged PFS and increased OS and the objective response rate ✓ The incidence of adverse events was lower with T-DXd	T-DXd increased PFS, improved OS and reduced the risk of death	[160]
**TNBC Therapy**				
Understand the benefits and risks of including anthracyclines, and the comparative benefits of different anthracycline–taxane regimens	Patient-level meta-analysis	✓ Anthracycline plus taxane reduced the rate and mortality of breast cancer ✓ Adding anthracycline to chemotherapy reduced disease recurrence	Adding anthracyclines to a taxane regimen increases the benefit by reducing the recurrence and the risk of mortality	[163]
Assess the benefits and harms of platinum-based chemotherapy as adjuvant and neoadjuvant treatment in people with early triple-negative breast cancer	Systematic review	✓ Platinum-based neoadjuvant chemotherapy improved DFS and reduced mortality ✓ Platinum-based adjuvant chemotherapy improved DFS and increased OS	Platinum-based chemotherapy improved DFS and OS in both treatment modalities	[164]
Investigate the efficacy and adverse effects of low-dose capecitabine maintenance after standard adjuvant chemotherapy in early-stage TNBC	Randomized clinical trial in China	✓ Adding capecitabine reduced breast cancer recurrence and mortality ✓ It improved the 5-year DFS, OS and locoregional recurrence-free survival ✓ The most frequent adverse event was hand–foot syndrome	Adding capecitabine after standard chemotherapy improved DFS, OS and locoregional recurrence-free survival	[165]
Evaluate the efficacy and safety of atezolizumab plus nab-paclitaxel in patients with unresectable, locally advanced or metastatic TNBC	IMpassion130 randomized, double-blind clinical trial	✓ PD-L1-positive tumors treated with atezolizumab plus nab-paclitaxel increased the OS and prolonged PFS ✓ PD-L1-negative tumors treated with atezolizumab plus nab-paclitaxel did not have any improvement ✓ Most common adverse event was neutropenia	Atezolizumab benefits OS and PFS, although its efficacy is confined to PD-L1-possitive tumors	[168]
Evaluate the efficacy of pembrolizumab as first-line therapy for patients with PD-L1-positive metastatic TNBC	International, open-label, multicohort clinical trial	✓ PFS and OS were longer with pembrolizumab in patients with disease-free interval higher than 12 months ✓ The objective response rate was higher when the disease-free interval was higher than 12 months ✓ The most common immune-related adverse event was hypothyroidism	Higher response rate to pembrolizumab as first-line treatment benefits patients with metastatic TNBC, especially those with PD-L1-positive tumors	[169]

### 4.4. Therapy Resistance

Breast cancer therapies, despite their effectiveness, often face significant challenges due to tumor heterogeneity, treatment resistance and toxicity that may reduce therapy efficacy and enhance disease recurrence [68,71]. As tumor heterogeneity pertains to a wide range of genetic and epigenetic modifications, it may difficult disease diagnostic, lead to treatment resistance and reduce prediction of prognosis resulting in treatment inefficacy, metastasis and disease recurrence [173]. Technology advancements allowed physicians to better understand this variability with single cell transcriptomics, also known as single-cell RNA sequencing. This method constructs the genetic profile of each individual cell within each tumor of the patient, providing a clear characterization of the tumor heterogeneity mechanisms, interaction between cells and signaling cascades related to poor therapeutic response or prognosis [174]. Although single-cell RNA sequencing proved to be a thorough molecular technique in explaining the oncologic mechanisms involved in tumor heterogeneity, the processes that lead to intratumor heterogeneity are still far from being understood and further research is needed as it represents an obstacle to the efficacy of targeted therapies [175].

A major source of tumor heterogeneity is cancer metastasis that originates from genetic mutations and epigenetic modifications that promote cancer cells to migrate and colonize other organs, predominantly bones, liver, brain and lungs. Additionally, the immune cells surrounding the primary tumor exert a selective pressure that creates treatment resistance and stimulates cancer metastasis [176,177]. Unraveling these interactions and modifications with single-cell RNA sequencing helps pave the way for the development of patient-tailored therapies with great efficacy potential using a combinatory drug approach. Recent studies using single-cell RNA sequencing to explore immune cell clusters on tumor microenvironment (TME) that modulate tumor heterogeneity revealed that each cell has a unique molecular signature within the same tumor and that its presence or absence can regulate different responses to breast cancer therapies and aid physicians in treatment selection [178,179].

Endocrine therapy resistance involves ER alterations, gene mutations, dysregulated expression patterns of co-regulatory proteins and alterations in signaling pathways and cell cycle modulators [180,181,182]. The ER molecule is a key component in ER-positive breast cancer and the main target of endocrine therapies; thus, ER alterations or mutations are a major cause of therapy resistance. The loss of ER expression prevents therapeutic drugs from targeting cancer cells, and this is strongly attributed to methylation of ER gene promoter region [183]. Manouk K.B. et al. confirmed that, in fact, ER expression can be regulated by DNA methylation, decreasing its levels of expression [184]. A small cohort of patients with ESR1-positive methylation and ESR1-negative methylation revealed that this alteration is related to reduced survival and increased therapy resistance [185]. Additionally, mutations in ESR1 result in ligand-independent constitutive activation and in the activation of multiple sets of target genes, increasing metastasis [186]. The hot spot mutations of this gene are D538G (18.6%), Y537S (13.1%), Y537N (8.8%) and E380Q (3.3%) [187]. In addition to therapy resistance caused by ESR1 mutations, evidence shows that PIK3CA gene mutations have a major role in the development of endocrine therapy resistance and that insulin-like growth factor 1 interacts with ER, using the PIK3/AKT/mTOR pathway to cause estrogen-independent activation of ER and therapy resistance [188,189].

Anti-HER2 therapy resistance can be attributed to changes in HER2 expression, loss of PTEN or overexpression of p95HER2. The upregulation of this truncated form of HER2 lacks an extracellular domain where drugs would bind, rendering therapy resistance [190]. Overexpression of HER2 is the main trigger for HER2-positive breast cancer, but the loss of HER2 is the cause of therapy resistance. This can be explained by a shedding mechanism that cleaves the extracellular and the intracellular juxtamembrane region that also induces p95HER2 production [191]. Recent findings report the existence of trastuzumab resistance whenever p95HER2 expression was elevated, and it was noteworthy that therapy resistance was significantly associated with lymphatic invasion, perinodal invasion, nodal involvement and metastasis [192]. Additionally, the upregulation of CMTM6 causes trastuzumab resistance and promotes breast cancer proliferation by increasing HER2 expression through the manipulation of downstream MAPK and PI3K/AKT/mTOR signaling pathways [193]. Furthermore, breast cancer cells may also be resistant to ADC due to changes in the routes of HER2 internalization, alterations in antibody–drug ratios may affect ADC efficacy, and mutations in PIK3CA gene and loss of PTEN may disrupt PI3K/AKT/mTOR signaling pathway, decreasing the effectiveness of ADC treatment [194,195].

TME is a complex and dynamic network of cellular and non-cellular components, including mesenchymal cells, cancer-associated fibroblasts (CAF), tumor-associated macrophage and endothelial cells within a tumor. It is an active participant in tumor onset and progression and influences treatment resistance in TNBC [196]. Zhaoze G. et al. found that in vitro and in vivo models of breast CAFs promoted cancer growth and radioresistance due to high levels of IL-6 secretion from these cells by the modulation of STAT3 [197]. In addition, tumor-associated macrophages contribute to therapy resistance by secreting inflammatory molecules, such as IL-6, TNF-α and CCL18, which suppress the function of T cells and by reprogramming the metabolic system to induce tumorigenesis [198]. On another note, heat shock protein beta-1 (HSPB1) exerts antioxidant and anti-apoptotic effects, but the overexpression of this protein promotes chemotherapy resistance through the NF-κB signaling pathway and provokes epithelial–mesenchymal transition, thereby facilitating cancer cells migration and invasion [199]. ATP-binding cassettes (ABC) transporters use ATP to efflux molecules, such as therapeutic drugs, present within cell; thus, a great number of ABC decrease therapeutic effects and even confer therapy resistance [200]. Additionally, recent findings explored the impact of two different enzymes on breast cancer chemoresistance. The upregulation of paraoxanase-2 decreased TNBC cell’s susceptibility to doxorubicin, 5-Fluorouracil and cisplatin leading to cell proliferation and therapy resistance, while nicotinamide N-methyltransferase upregulation leads to SIRT1 stabilization by preventing its degradation, which increases its deacetylation activity promoting cell survival and therapy resistance [201,202].

Overcoming resistance remains a major challenge in breast cancer treatment, necessitating further research into combinatory therapies, novel drug development, and personalized treatment strategies to increase long-term patient outcomes. Recent studies using CDK4/6 inhibitors in combination with therapeutic drugs showed an improvement in endocrine resistance caused by ESR1 mutations, reducing treatment failure [203,204]. Moreover, targeting PI3K/AKT/mTOR signaling pathway with a combination therapy of PI3K inhibitors and trastuzumab to treat HER2-positive breast cancer can ameliorate resistance and restore apoptotic effects [205]. Additionally, a clinical trial using a tyrosine kinase inhibitor (tucatinib), together with trastuzumab and capecitabine, improved OS and PFS, suggesting overcoming therapy resistance [206]. A study on a combinatory strategy of pembrolizumab plus chemotherapy on TNBC revealed that the conjugation was more effective than using only chemotherapy to treat patients, significantly improving the OS of these patients [207]. Furthermore, a meta-analysis on PARP inhibitors on patients with germline BRCA1/2 mutations revelated that this targeted therapy increases the overall response to treatment overcoming chemoresistance [208]. Another study using PARP inhibitors (olaparib) and CDK4/6 inhibitors (palbociclib) demonstrated that this combinatory strategy was an effective approach to overcome cellular olaparib resistance in comparison to olaparib monotherapy, enhancing treatment response [209].

Despite clinical efforts for developing efficient treatment for primary tumors, metastatic breast cancer therapy does not always respond to treatment due to tumor heterogeneity that varies according to cancer subtype, metastatic site and patient, leading to therapy resistance [176]. However, recent research on using targeted therapies and combinatory approaches elucidates an efficient strategy to improve the survival of patients with metastatic disease and reduce resistance. A study comparing the use of fulvestrant plus capivasertib with fulvestrant plus placebo on patients with metastatic ER-positive and HER2-negative breast cancer patients indicates that capivasertib improves OS and PFS [210]. Another study using pyrotinib, trastuzumab and docetaxel found that this three-drug approach ameliorated PFS in comparison to a placebo group of patients with metastatic HER2-positive breast cancer [211]. A cohort of patients with metastatic TNBC treated with atezolizumb and nab-paclitaxel had higher PFS and higher OS in comparison to patients treated with placebo and nab-paclitaxel [212]. Contradictory, recent research on patients with metastatic TNBC did not find significant differences in OS between patients that received atezolizumb and chemotherapy in comparison to those that received a placebo plus chemotherapy [213]. This may be explained by the aggressive and heterogenous nature of TNBC that entails significant genetic, molecular and cellular variability in comparison to other breast cancer subtypes, making it more difficult to treat. Therefore, further research must be conducted to better understand how to respond to metastatic TNBC.

To this extent, numerous studies have positively correlated combinatory therapy strategies to treatment response over monotherapy. The employment of this approach offers several benefits, particularly in overcoming therapy resistance. By targeting multiple pathways simultaneously, combinatory approaches reduce the likelihood of cancer cells developing resistance to a single drug [214]. Additionally, combining targeted therapies with chemotherapy or immunotherapy can enhance tumor cell death while minimizing off-target effects [215]. This strategy also helps address tumor heterogeneity by targeting different subpopulations within a tumor, thereby reducing the chances of relapse [216].

## 5. Conclusions

Breast cancer remains a major public health challenge due to its molecular heterogeneity and therapy resistance. However, a comprehensive analysis of the molecular subtypes of breast cancer and their respective therapies helps physicians in treatment selection by reducing complicated side effects, therapeutic resistance and improving patient outcomes.

Advancements in technology have allowed the development of single-cell transcriptomic analysis to identify different cell populations within a tumor, providing a clear insight of tumor heterogeneity that often leads to therapy resistance. Nevertheless, despite significant efforts in managing advanced breast cancer, the heterogenous nature of the disease continues to be a major challenge, as it poses a key obstacle in promoting resistance to targeted therapies. Therefore, understanding tumor characteristics is crucial for the development of personalized therapeutic strategies and the continuous adaptation of therapeutic approaches.

Furthermore, to the best our knowledge, this is the first review that highlights the importance of combinatory therapies over monotherapies in overcoming therapy resistance in every breast cancer subtype, providing an extended explanation on resistance mechanisms and proposing the utilization of multi-targeted strategies to target multiple pathways involved in therapy resistance to reduce disease recurrence.

Addressing these limitations of tumor heterogeneity and treatment resistance by developing an integrative approach with molecular profiling, single-cell transcriptomic and combinatory therapies pave the way for the creation of multimodal treatments where cancer management is effective and patient-centered, contributing to great advancements in precision oncology. Therefore, future research should focus on validating these findings into clinical practice and explore novel drug combinations to target adaptive resistance mechanisms and technological enhancement to increase drug response prediction and personalized treatment selection, as well as to help optimize treatment regimen in real time.

## Abbreviations

The following abbreviations are used in this manuscript:

ABC	ATP-Binding Cassette
ADC	Antibody-Drug Conjugate
AI	Aromatase Inhibitors
AIB1	Amplified in Breast Cancer 1
AKT	Protein Kinase B
Ap1	Activator Protein 1
BL1	Basal-Like 1
BL2	Basal-Like 2
BMI	Body Mass Index
BRCA1	Breast Cancer Associated Gene 1
BRCA2	Breast Cancer Associated Gene 2
CAF	Cancer-Associated Fibroblasts
CCL18	Chemokine (C-C motif) Ligand 18
CL	Claudin-Low Type
CMTM6	CKLF-like MARVEL Transmembrane Domain-Containing 6
CTLA-4	Cytotoxic T-Lymphocyte-Associated Antigen 4
DCIS	Ductal Carcinoma In Situ
DFS	Disease-Free Survival
E1	Estrone
E2	Estradiol
E3	Estriol
E4	Estetrol
EGFR	Epidermal Growth Factor Receptor
ER	Estrogen Receptors
ERE	Estrogen-Responsive Elements
ERK1	Extracellular Signal-Regulated Kinase 1
ERK2	Extracellular Signal-Regulated Kinase 2
GDP	Guanosine Diphosphate
GRB2	Growth Factors Receptor-Bound Proteins 2
GTP	Guanosine Triphosphate
HER2	Human Epidermal Growth Factor Receptor 2
HSPB1	Heat Shock Protein Beta-1
IBC	Inflammatory Breast Cancer
ICI	Immune Checkpoint Inhibitors
IDC	Invasive Ductal Carcinoma
IL-6	Interleukin-6
ILC	Invasive Lobular Carcinoma
IM	Immunomodulatory
LAR	Luminal Androgen Receptor
LCIS	Lobular Carcinoma In Situ
M	Mesenchymal-Like
MAPK	Mitogen-Activated Protein Kinase
MEK 2	Mitogen-Activated Protein Kinase Kinase 2
MEK1	Mitogen-Activated Protein Kinase Kinase 1
MRI	Magnetic Resonance Imaging
MSL	Mesenchymal Stem-Like
MTA1	Metastasis-Associated Protein 1
mTOR	Mammalian Target of Rapamycin
NCOR	Nuclear Receptor Corepressor
NFκB	Nuclear Factor-κB
OS	Overall Survival
pCR	Pathological Complete Response
PD-1	Programmed Death Receptor-1
PD-L1	Programmed Death Receptor-Ligand 1
PFS	Progression-Free Survival
PI3K	Phosphatidylinositol 3-Kinase
PR	Progesterone Receptors
PTEN	Phosphatase and Tensin Homolog
RANK	Receptor Activator of Nuclear Factor κB
RANKL	Receptor Activator of Nuclear Factor κB Ligand
RAS	Rat Sarcoma
SERM	Selective Estrogen Response Modulator
SOS	Son Of Sevenless
SP1	Specificity Protein 1
SRC 1	Steroid Receptor Coactivor 1
STAT3	Signal Transducer and Activator of Transcription 3
TAM	Tamoxifen
T-DM1	Trastuzumab Emtansine
T-DXd	Trastuzumab Deruxtecan
TME	Tumor Microenvironment
TNBC	Triple-Negative Breast Cancer
TNF-α	Tumor Necrosis Factor Alpha
